# Design, Formulation and Physicochemical Evaluation of Clotrimazole Chewing GumDesign, Formulation and Physicochemical Evaluation of Clotrimazole Chewing Gum

**DOI:** 10.31661/gmj.v10i0.1084

**Published:** 2021-06-11

**Authors:** Abolfazl Aslani, Shekofeh Karbasizadeh Esfahani

**Affiliations:** ^1^Department of Pharmaceutics, School of Pharmacy and Novel Drug Delivery Systems Research Center, Isfahan University of Medical Sciences, Isfahan, Iran; ^2^ Novel Drug Delivery Systems Research Center, Isfahan University of Medical Sciences, Isfahan, Iran

**Keywords:** Clotrimazole Chewing Gum;, Oral Candidiasis;, Oral Mucosal Drug Delivery

## Abstract

**Background:**

Oral candidiasis is widespread in the patients with immunodeficiency diseases. Chewing gums are considered as mobile drug delivery systems that affected locally or systemically via the oral cavity. This study aimed to develop and evaluate the formulation of clotrimazole chewing gums for patients having oral candidiasis.

**Materials and Methods:**

Fourteen formulations (F) were designed by Design-Expert, version 7. These formulations were different in the amount of gum bases and sweeteners. Gum bases of elvasti, 487, stick and fruit C were heated up to 70°C. Clotrimazole powder, sugar, liquid glucose, glycerin, mannitol, xylitol, and maltitol as well as different flavoring agents were added to the gum bases at 40°C. Content and weight uniformity, organoleptic properties evaluation, releasing the active ingredient in the phosphate buffer pH, 6.8 and taste evaluation were analyzed by Latin square analysis. Also, the mechanical test was done on F_13_ and F_14_ formulations.

**Results:**

F_14_ was the best formulation in terms of organoleptic properties. This formulation had suitable size, hardness, softness, and lack of adhesion to teeth. F_14_ formulation released 89% and 97% of clotrimazole after 30 and 45 minutes, respectively. F_14_ content uniformity and weight variations were 9.83±0.086 mg and 1.14±0.09 g, respectively. F_14_ evaluation of mechanical properties showed Young’s modulus about 0.32 MPa, and yield point occurred at the stress of 0.599 MPa and strain of 4.1%.

**Conclusion:**

F_14_ was chosen according to its physicochemical and organoleptic properties. F_14_ had adequate hardness, lack of adhesion to the teeth, suitable size, and best drug release. Tutti Frutti was a proper flavoring agent for clotrimazole gum formulations.

## Introduction

Candida albicans is a pathogen responsible for fungal infections affecting the buccal cavity. When the immune system is exposed to changes including weakened immune system, *c. albicans* causes the incidence of lesions in the buccal cavity. The predisposing factors for candidiasis include diabetes, kidney discomfort, dry mouth, trauma, buccal pH, and buccal plaques [1]. Studies have shown that the prevalence of Candida species in the mouth of patients with thalassemia is higher than that of healthy people, while *c. albicans* has the highest percentage. Splenectomy, increased blood iron and serum ferritin can increase candida colonization in the mouth [2]. According to many studies throughout the world, *c. albicans* is the most common species of Candida in developing the disease and is the most common species isolated from HIV patients [3]. Taking medications that affect the immune system, such as chemotherapy drugs and antibiotics, may also eliminate the microflora so that the bacteria naturally found in the oral cavity are reduced to their normal levels. As a result, the fungal population increases in the mouth, followed by oral candidiasis [4]. Nystatin was discovered in 1951 and amphotericin B in 1956 for the treatment of buccal candidiasis. These two antibiotics are not absorbed through the gastrointestinal tract. Miconazole, which is an imidazole, can be used as a topical oral treatment; however, this form of usage is limited because of the fear of unwanted side effects such as vomiting and diarrhea. Other drugs applied in oral candidiasis treatment include clotrimazole and ketoconazole [5]. Clotrimazole is a synthetic antifungal medication. This medicine is crystalline powder, odorless, and yellowish-white. Clotrimazole is insoluble in water, but it is easily soluble in alcohol and polyethylene glycol 400 [6]. The clotrimazole is a synthetic derivative of imidazole. Azole compounds inhibit the lanosterol 14-alpha-demethylase (sterol 14α-demethylase) enzyme. This enzyme converts lanosterol to ergosterol by removing the 14-α-methyl group. Sterol 14α-demethylase is a cytochrome P-450 dependent enzyme, and the fungal cytoplasmic membrane plays an important role in the synthesis of ergosterol. Inhibition of 14α-demethylase sterol impairs the permeability of the fungal cytoplasmic membrane, which increases the permeability of the membrane. As a result, the intracellular elements of the fungus are reduced [7]. Clotrimazole is effective in treating more fungal infections, and is applied in the treatment of Tinea pedis, Tinea cruris, Tinea versicolor, Tinea corporis, skin candidiasis, vulvovaginal candidiasis and oropharyngeal candidiasis, as well as the prevention of oropharyngeal candidiasis in immunocompromised patients [8,9].Several formulations, such as topical cream/ointment/solution, oral tablet/lozenge, and vaginal cream/pessary have been produced for clotrimazole. Medicinal chewing gums can release their medication slowly in the saliva [10]. Drug delivery through medicinal chewing gums has some benefits, including the higher capacity of chewing gum compared with other forms of medication [11]. The medicinal chewing gums can create the long-lasting topical effect by controlling the release rate of active components [12]. The use of chewing gum form is easy and requires no water [10]. The chewing gums, especially xylitol chewing gums, reduce the severity and frequency of dental caries by increasing pH of plaque [13]. The chewing gum increases the saliva secretion and prevents dry mouth, resulting in better healing and immunity of the oral cavity [14]. The formulation of medicinal chewing gums consists of active ingredients, chewing gum bases, water-insoluble components, including elastomers, fillers, waxes, fats, resins, and emulsifiers, and water-soluble components, including softeners, sweeteners, flavoring agents, antioxidants and colorants [15]. The release rate of the active ingredients of formulation in the medicinal chewing gums depends on the physicochemical characteristics of the active ingredients, the active ingredient ratio, contact time, and individual chewing properties [16,17]. Regarding the mentioned issues, there is a shortage in the dosage form of clotrimazole, which has a topical oral effect in the pharmaceutical market. The purpose of this project is to design, formulate and evaluate physicochemical properties of clotrimazole chewing gum used in the treatment of oral candidiasis. Clotrimazole chewing gums with continuous release cause fungicidal and fungistatic effects.

## Materials and Methods

### Materials and Reagents

Clotrimazole was purchased from Tehran-Daru Pharmaceutical Company (Tehran, Iran). Gum bases such as Elvasti, Fruit C, 487 and Stick were obtained from Gilan Ghoot Company (Rasht, Iran). Flavoring agents such as eucalyptus, menthol, cinnamon, and banana were from Goltash Company (Isfahan, Iran) and flavoring agents of cherry and tutti-frutti were provided from Farabi Pharmaceutical Company (Isfahan, Iran). Materials such as maltitol, xylitol, mannitol, glycerol, aluminum chloride, potassium acetate, potassium dihydrogen phosphate, sodium hydroxide, ethanol, chloroform, and polyethylene glycol 400 (PEG 400) were prepared from Merck Company.

### Clotrimazole Standard Curve in Phosphate Buffer

Clotrimazole powder is insoluble in phosphate buffer. Ten mg of clotrimazole powder was prepared in a 100-ml volumetric flask, and the volume reached to 100 ml using the solvent system, containing 30% PEG 400, 40% phosphate buffer, pH 6.8, and 30% ethanol and shake well to be dissolved totally. Then, 1, 2, 4, 6, 8 and 10 ml of this solution were removed, respectively, and reached 100 ml in the 100-ml volumetric flask. In this way, the concentrations of 1, 2, 4, 6, 8 and 10 μg/ml were prepared. The wavelength of maximum absorbance (λmax) was determined using the concentration of 6μg/ml. The UV-visible spectrophotometer (Shimadzu, UVmini-1240) was then adjusted at this wavelength (λmax) to measure the absorbance of the concentrations. In this experiment, three specimens were made from each concentration and the testing was repeated for three consecutive days. The standard curve was drawn after obtaining the mean absorbance, and the linear equation was achieved.

### Clotrimazole Standard Curve in Ethanol

To draw the clotrimazole standard curve in ethanol, 10 mg of clotrimazole powder was weighed and reached 100 ml by ethanol in the 100-ml volumetric flask and dissolved well. Then, 1, 2, 4, 6, 8 and 10 ml of this solution were removed, respectively, and reached 100 ml in the 100-ml volumetric flask. In this way, the concentrations of 1, 2, 4, 6, 8 and 10 μg/ml were prepared. The wavelength of maximum absorbance (λmax) was determined using the concentration of 6μg/ml, which was 259 nm. In this experiment, three specimens were made from each concentration, and the testing was repeated for three consecutive days. The standard curve was drawn after obtaining the mean absorbance, and the linear equation was achieved.

### Preparation of Clotrimazole Chewing Gum

The Design Expert Software d-optimal model was used to determine the best ratio of chewing gum bases in formulations. In the d-optimal model for chewing gum of clotrimazole, the amount of drug-releasing was evaluated over the course of 30 minutes of chewing relative to the amount of the chewing gum base. In this study, the range of hard bases (Elvazti, 487) and soft bases (Stick, Fruit C) was selected between 60 and 80 milligrams (Table-1). >The variable of the base was evaluated in two levels (hard and soft) as well as the amount of drug release within 30 minutes. According to the data inserted to the Design-Expert program, based on these two variables, nine formulations were presented by the program to us for review (E_1_ –E_9_), and drug release according to the bases was measured over 30 minutes, and the rate of drug release in mg with each base ratio was obtained during 30 minutes (Table-1). Then, after the measurements, the Design-Expert program was asked to provide the optimum formulation(s) for drug release within 30 minutes. Design-Expert software proposed optimum formulation (OE) with the predicted percent of release, after formulated this formulation, obtained the percent of release compared with an estimated percent of release and calculated error percentage, so OE ignored by high error percentage (Table-2). Therefore, E_1_ and E_2_ were selected as the main formulations to continue the study. Two formulations were selected with the base ratios of 60:60 (E_1_) and 70:70 (E_2_), respectively, as optimum formulations. According to the results, these two formulations were selected by Design-Expert program as the optimum formulation. The formulation with the base ratio of 60:60 (E_1_) released 94% of clotrimazole over 30 minutes, and the formulations with the base ratio of 70:70 (E_2_) liberated 91% of clotrimazole in 30 minutes. Finally, the formulation accepted after the examination was the hard/soft base ratio of 70:70 (E_2_) because this formulation had better appropriate organoleptic properties compared to others. According to Table-3, the certain quantities of each base in the chewing gum formulations were weighed to form the clotrimazole chewing gum, heated inside the mortar and placed in water bath at 70°C to soften the gum bases. Then, the clotrimazole powder was pulverized, and then levigated in liquid glucose and glycerin. Other sweeteners were also added to this mixture and mixed well. Then the resulting mixture was added to the softened gum base. Finally, when the temperature of the gum mixture decreased to 40°C, the desired flavor was added to it and mixed. After complete mixing, the chewing gum was removed, placed on a glass plate and cut to smaller pieces in proper thickness and size, placed in room temperature for 48 hours and then packed. F_1_-F_4_ formulations were ignored according to what was explained by Design-Expert results.

### Weight Uniformity Test

Twenty chewing gums were randomly selected among the chewing gums, and the mean weight of 20 samples was determined. The chewing gums weighed should not differ with the mean weight over 5% [18].

### Content Uniformity Test

To study the content uniformity of the chewing gums, ten chewing gums were randomly selected from the chewing gums produced, and each chewing gum was dissolved in 25 ml of ethanol [19]. Ten ml of this solution was centrifuged at 3200 rpm. Then, one ml of clear supernatant solution was removed and reached 10 ml using ethanol. The absorbance of this solution was measured at a wavelength of 259 nm by UV-visible spectrophotometer. This experiment was performed in three replications. The placebo chewing gum (without medication) was also dissolved in ethanol, and its absorbance was measured by the same method and subtracted from the absorbance of each chewing gum.

### Evaluation of Organoleptic Properties of Clotrimazole Chewing Gum

Organoleptic properties including taste stability, appropriate volume, and size, non-adhesion to teeth, hardness, and softness were studied [16]. To evaluate the organoleptic properties of clotrimazole chewing gum, each formulation was given to 10 healthy volunteers, and their opinion was asked about the hardness and softness, the volume of the chewing gum, and the non-adhesion to the teeth and taste. It should be noted that volunteers were unaware of the differences in formulations. The formulations were presented to the volunteers using the Latin Square method. Organoleptic properties of clotrimazole chewing gum were scored based on the Lickert scale by volunteers so that each property received a score of 1-5.

### Clotrimazole Release From Clotrimazole Chewing Gum 

The European Pharmacopeia (EP) of the jaw movement simulator [20] has been suggested to investigate drug release from the clotrimazole chewing gum. A piece of chewing gum was placed in the device chamber. The chewing machine was started at 37± 0.5°C and with the volume of the dissolution space of 50 ml phosphate buffer, pH 6.8 and the rate of 60 strokes per minute. The sampling was done from the chamber at 5, 10, 15, 30 and 45 minutes. In each sampling, 1 ml was removed from the chamber and 1 ml fresh phosphate buffer, pH 6.8, was added to the chamber at 37±0.5°C. The placebo chewing gum (without medication) was also placed in the chewing machine and was sampled at the mentioned intervals. The samples reached the volume using the solvent system containing, 30% PEG 400, 40% phosphate buffer, pH 6.8, and 30% ethanol. The absorbance of the clotrimazole was measured by a UV-visible spectrophotometer (Shimadzu, UVmini-1240) at the maximum wavelength of 245 nm. This experiment was performed in three replications. To record the results of this test, the absorbance of the placebo chewing gum samples was subtracted from the absorbance of clotrimazole chewing gums.

### Evaluation of Different Flavoring Agents

The taste of clotrimazole chewing gum was evaluated by panel test. The selected formulations in terms of organoleptic properties were prepared in six different flavors of menthol, tutti-frutti, cinnamon, cherry, eucalyptus, and banana, and then were presented to 20 healthy volunteers. Total scores obtained by the candidates were calculated for each formulation. In order to determine the best flavoring in the last stage, 30 volunteers were selected from among the two selected flavorings of the previous stage, which received the highest score.

### Evaluation of the Mechanical Properties of the Prepared Chewing Gum

In the tensile test, each chewing gum sample was subjected to tensile testing by a universal testing machine (STM, Santam, Iran) in the form of a dumbbell with 15 mm width and 4 mm thickness at ambient temperature. The gauge space was 50 mm, and the tension velocity of the samples was 50 mm/min.

## Results

### Clotrimazole Standard Curve in Phosphate Buffer

The wavelength of maximum absorbance (λmax) was determined 245 nm. In addition, the linear equation obtained from the curve drawn was y=0.1556x+0.0011 with R²=0.999. Clotrimazole Standard Curve in EthanolThe wavelength of maximum absorbance (λmax) was achieved 259 nm. Moreover, the linear equation obtained from the curve drawn was y=0.3265x-0.0022 with R²=0.999.

### Content Uniformity Test

The content uniformity test was performed for ten chewing gums from two superior formulations of F_13_ and F_14_. The mean content uniformity for F_13_ and F_14_ formulations was 9.82±0.082 and 9.83±0.086, respectively.

### Weight Uniformity Test

According to United States Pharmacopeia (USP), the weighed chewing gums should not differ more than 5% from the mean weight. The mean weight of 20 chewing gums was determined from each of the superior formulations of F_13_ and F_14_, calculated to be 1.14±0.86 g and 1.14±0.09 g, respectively.

### Results of Organoleptic Properties

The results of the study of organoleptic properties are presented in Table-4. This table indicates the volume of chewing gum mass, softness and stiffness, and non-adhesion to the teeth during chewing and their flavors. F_13_ and F_14_ were selected as the superior formulations in terms of organoleptic properties.

### Release Test for Chewing Gum Formulations

**Figure 1:**
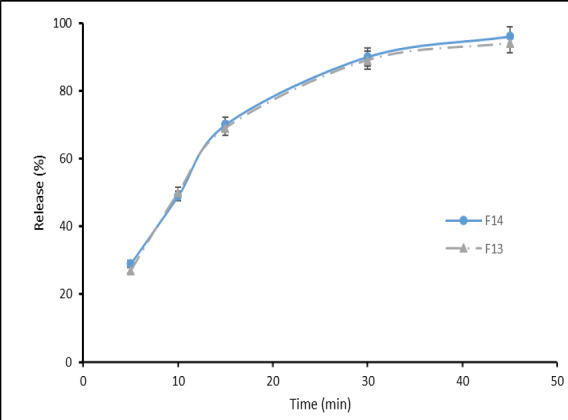
In vitro release of clotrimazole chewing gum in pH, 6.8 phosphate buffer at 37°C

**Figure 2:**
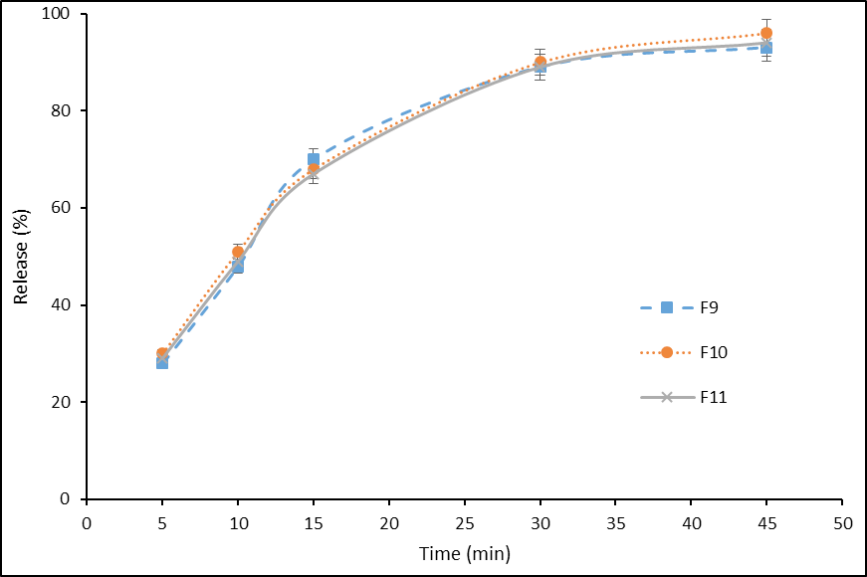
In vitro release of clotrimazole chewing gum formulations with various sweeteners in pH, 6.8 phosphate buffer at 37°C

**Figure 3:**
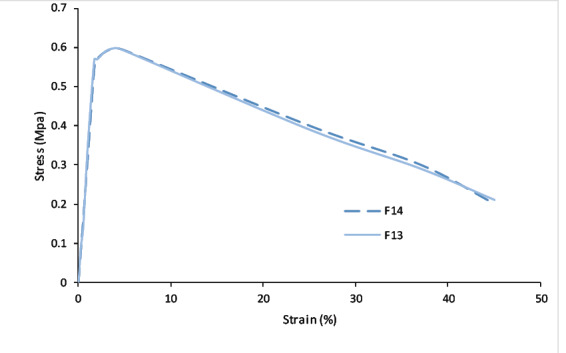
Tensile behavior of F_13_ and F_14_ formulations of clotrimazole chewing gum (same gum bases)

The superior formulations of F_13_ and F_14_ were selected to examine the clotrimazole release from the clotrimazole chewing gum, as well as the formulations of F_6_- F_12_, were selected to study the effect of used sweeteners (maltitol, mannitol, xylitol) on the drug release from the chewing gum base. It should be noted that after 30 minutes, 87% and 89% of clotrimazole and after 45 minutes, 94% and 97% of the drug, were released from F_13_ and F_14_ formulations respectively (Figure-1). Also, after 30 minutes, about 89% of clotrimazole and after 45 minutes, around 95% of the drug was released from F_9_, F_10_ and F_11_ formulations (Figure-2). The Best Flavoring AgentsThe results of the panel test performed on the use of various flavoring agents are given in Table-5. Tutti-frutti and eucalyptus flavoring agents were selected in the first phase and in the second phase, tutti-frutti was chosen (Table-6). The Mechanical Properties of Clotrimazole Chewing Gum The stress-strain diagram of tensile test for F_13_ and F_14_ formulations is shown in Figure-3. The Young’s modulus of both formulations is about 0.32 MPa. The yield points of formulations are about 0.599 MPa in stress and 4.1% in strain.

## Discussion

*c. albicans* is the oral fungal infection that can grow in patients with the weakened immune system. Clotrimazole is one of the topical treatments for candidiasis. It is effective in the treatment of most fungal infections. The purpose of this study was to formulate and investigate the physicochemical properties of oral clotrimazole chewing gum. This chewing gum gradually releases its drug content and creates the proper topical effect.According to the content of the two F_13_ and F_14_ formulations, both of them in terms of content uniformity was within the acceptable range of the EP (85-115% of the mean content). Similarly, the chewing gum samples for weight uniformity were consistent with the EP. None of the 20 samples of the randomly selected chewing gum was different over 5% with the EP index. Among the F_1_ to F_5_ formulations in terms of hardness and softness of the chewing gum and non-adhesion to the teeth, the F_3_ formulation had the highest amount of hard bases (Elvazti and 487) and was harder compared to other formulations. The F_4_ formulation contained the highest amount of soft bases (fruit C and stick) and was softer than other formulations. The F_1_ and F_2_ formulations were somewhat hard. The formulation F_5_, which had equal amounts of bases, showed good hardness compared to the other four formulations (Table-3,Table-4), so the equal ratio of the bases were chosen to continue the work. The different proportions of various sweeteners were added to these bases to select the best taste and organoleptic properties. The addition of mannitol partly softens the chewing gum base and makes the chewing gum taste inappropriate. It also produced less sweetness than maltitol. On the other hand, the addition of xylitol gave a pleasant flavor and mouth cooling to chewing gum, and created the perfect sweetness for chewing gum, which is a good alternative to sweet sugar and reduces tooth decay and dental plaque. Of the six different flavors including menthol, tutti-frutti, cinnamon, cherry, eucalyptus and banana, the eucalyptus and tutti-frutti flavorings were selected in the first phase (Table-5), and tutti-frutti was chosen as the top flavoring in the final phase (Table-6). In a study performed by Aslani and Jalilian, the volunteers selected the cinnamon flavor as the best one [22]. Also, in another study conducted by Aslani and Rafiei to make the nicotine chewing gum, cherry and eucalyptus flavors were selected as the best flavors [16]. To make green tea chewing gum, another study was performed by Aslani et al. the mint and cinnamon flavors were selected by volunteers [23]. Furthermore, in the study by Aslani et al., to make Aloe Vera chewing gum, the mint was chosen as the best flavoring agent [21].The clotrimazole release from the chewing gum base of the superior formulations of F_13_ and F_14_ was 94% and 97% after 45 minutes of chewing, respectively. According to Figure-1, the release rate of F_13_ and F_14_ formulations at 5, 10, 15, 30, and 45 minutes is approximately identical and has no much difference. The analysis of the release results for F_9_, F_10_, and F_11_ formulations showed that the sweetener type did not significantly affect the release rate. This slight difference for release goes back to the nature of the sweetener. It should be noted that the placebo chewing gum (without medication) had no absorbance at this wavelength.In the study performed by Aslani and Jalilian, the release of caffeine in chewing gum at 10, 20 and 30 minutes were reported to be 55%, 78%, and 89%, respectively [22].Also, in another study by Aslani and Rafiei, the release of nicotine from 2 to 4 mg chewing gums at 20 min was reported to be 83% and 79%, respectively [16].Furthermore, in the study by Aslani et al., to make Aloe Vera chewing gum over than 90% of carbohydrate was released after 30 min [21].To evaluate the results of the mechanical properties of chewing gum (Figure-3), the superior formulations of F_13_ and F_14_ showed linear elastic behavior initially due to the stress applied by the device, and then entered the nonlinear region with increasing strain, next reached to yield point with increasing strain. In the following, the samples exhibited plastic behavior with an increase in applied strain, and entered the whitening region and tear occurred before reaching the hardening region due to stress.

## Conclusion

The results of this study showed that chewing gum could be a very suitable form for buccal drug delivery of clotrimazole. The formulation F_14_ had the best organoleptic properties and had a proper drug release within the prescribed time. Also, the tutti-frutti was chosen as the best flavoring agent.

## Acknowledgment

This study was supported by grants from Isfahan University of Medical Sciences as a thesis research project (No. 394801).

## Conflict of Interest

Authors declare have no conflict of interests.

**Table 1:** Formulations of Clotrimazole Chewing Gum Designed by Design-Expert Software with Hard and Soft Bases According to Drug Release Percent after 30 Minutes

**Formulations**	**Elvazti (mg)**	**487 (mg)**	**Stick (mg)**	**Fruit C (mg)**	**Release (%)**
E_1_	60	60	60	60	94
E_2_	70	70	70	70	91
E_3_	80	60	80	60	82
E_4_	60	80	60	80	81
E_5_	80	80	60	60	78
E_6_	60	60	80	80	84
E_7_	80	80	80	80	85
E_8_	70	70	60	60	73
E_9_	60	60	70	70	84

**Table 2:** Optimum Formulation that Proposed by Design-Expert Software

**Release (%)**	**Fruit C (mg)**	**Stick (mg)**	**487 (mg)**	**Elvazti (mg)**	**Formulation**
O	E	69.19	69	60.05	62	OE
73	89.25

**OE:** Optimum formulation that proposed by Design-Expert;** E:** Estimated release percent;** O:** Obtained release percent

**Table 3:** Formulations of 10 mg Clotrimazole Chewing Gum with Different Ingredients

**Formulations**	**Ingredients (mg)**
F_14_*	F_13_
**Gum bases**	undefined
70	70
70	70
70	70
70	70
200	200
50	100
150	100
200	200
5	5
45	45
200	200

**Table 4:** The Averages of Scores for Organoleptic Properties of Clotrimazole Chewing Gum Formulations by Ten Volunteers

Organoleptic properties	Formulations												
F_1_	F_2_	F_3_	F_4_	F_5_	F_6_	F_7_	F_8_	F_9_	F_10_	F_11_	F_12_	F_13_	F_14_
Chewing gum volumea	2	2	2	2	2	3	3	3	3	3	3	3	3	3
Softness and Hardnessb	4	4	5	1	3	3	2	3	3	3	3	3	3	3
No adherencec	4	4	4	3	4	3	4	3	3	3	5	3	4	5
Tasted	1	1	1	1	2	1	2	3	1	3	3	3	4	5
													
													
													
													
													
													

a The bulk volume of gum was evaluated as Huge=5, much=4, right=3, little=2, very little=1. b The Softness/Hardness was evaluated as very hard=5, hard=4, suitable=3, soft=2, very soft=1. c The adherence to the teeth was evaluated as never adheres=5, rarely adheres=4, sometimes adheres=3,often adheres=2, always sticks=1. d The Taste was evaluated as excellent=5, good=4, fair=3, poor=2, very poor=1

**Table 5:** The Summations of Scores Allocated by 20 Volunteers for Superior Tastes of the Best Clotrimazole Chewing Gum Formulations

Formulations				
F_14_	F_13_	Flavoring Agent		
Median	Mean	Scores*	Median	Mean
3	3.4	69	3	3.2
5	4.6	93	5	4.5
4	3.8	77	2	3.7
3	2.3	46	3	2.2
4	4.6	92	4	4.5
2	3.4	69	3	3.3

*The taste was evaluated as excellent = 5, good = 4, fair = 3, poor = 2, very poor = 1

**Table 6:** The Summations of Scores Allocated by 30 Volunteers for Top Tastes of Best Clotrimazole Chewing Gum Formulations

Formulations						
F_14_	F_13_	Flavoring Agent				
Median	Mean	Scores*	Median	Mean	Scores*	
5	4.7	143	4	4.7	140	Tutti frutti
2	2.2	120	3	2.8	118	Eucalyptus

*The taste was evaluated as excellent = 5, good = 4, fair = 3, poor = 2, very poor = 1
